# Response of Microbial Community to Induced Failure of Anaerobic Digesters Through Overloading With Propionic Acid Followed by Process Recovery

**DOI:** 10.3389/fbioe.2020.604838

**Published:** 2020-12-11

**Authors:** Azin Khafipour, Elsie M. Jordaan, Daniel Flores-Orozco, Ehsan Khafipour, David B. Levin, Richard Sparling, Nazim Cicek

**Affiliations:** ^1^Department of Biosystems Engineering, University of Manitoba, Winnipeg, MB, Canada; ^2^Department of Animal Science, University of Manitoba, Winnipeg, MB, Canada; ^3^Department of Microbiology, University of Manitoba, Winnipeg, MB, Canada

**Keywords:** anaerobic digestion, propionic acid, methane inhibition, microbial dysbiosis, microbial diversity, biodigester failure, illumina sequencing

## Abstract

In order to effectively use microbial-based strategies to manage anaerobic digesters, it is necessary to distinguish between community shifts that are part of the natural dynamic of the system and shifts caused by environmental or operational disturbances. The objective of this research study was to evaluate the significance of changes in the microbial community of anaerobic digesters during failure in correlation to operational parameters such as an organic acid overload. Five continuously stirred 0.5 L reactors were set-up as semi-continuously-fed, mesophilic dairy manure digesters with a 30-day hydraulic retention time. After a 120-day stabilization period, two digesters were kept as controls, while the organic loading rates in the triplicate set were increased step-wise to ultimately provide a shock-load leading to failure using propionic acid spikes. Acidosis resulting in near cessation of biogas and termination of methane production occurred between 4 and 7 weeks, after which all the digesters continued to be fed only dairy manure. The shock loading of propionic acid led to an accumulation of mainly acetate and propionate, with low levels of iso-butyrate, butyrate, iso-valerate, and valerate. High-throughput Illumina sequencing of the V4 region of the bacterial and archaeal 16S rRNA gene in digester samples showed a significant change in the microbial community composition during propionic acid overload, followed by a return to the original composition with regular feedstock. Bacterial genera whose relative abundance decreased during the inhibition stage included *Sedimentibacter*, *Syntrophomonas*, *TSCOR003.O20*, and Marinilabiaceae, while the relative abundance of Lachnospiraceae, *Ruminococcus*, Mogibacteriaceae, *Pyramidobacter*, and *Bacteroides* increased. The relative abundance of dominant methanogens, *Methanosarcina* and *Methanobacterium*, although initially resistant, were decreased (from 91.71 to 12.14% and from 2.98 to 0.73%, respectively) during inhibition, while *Methanobrevibacter* and *Methanosphaera* that were prominent in the manure feedstock increased from 17.36 to 79.45% and from 0.14 to 1.12%, respectively. Shifts in bacterial and archaeal compositions, back to their pre-shock steady state after failure, highlight the digester’s microbial resilience and recovery potential.

## Introduction

Anaerobic digestion, a highly complex and dynamic process driven by a series of inter-linked biochemical transformations catalyzed by a consortium of microorganisms, is generally characterized by four basic phases: hydrolysis, acidogenesis, acetogenesis, and methanogenesis. The reactions in these phases influence one another by proceeding in consecutive and parallel steps, both spatially as well as temporally (Schoen et al., 2009; Jaenicke et al., 2011). In a stable anaerobic digester, the functionality of the microbial community would be expected to be redundant resulting in operational resilience. Some shifts in the microbial composition would be expected as a normal part of operation and digester performance, but not all steady states provide the same resilience to perturbations (De Vrieze et al., 2012). In some extreme cases, accidental spillage of chemicals (i.e., antibiotics, heavy metals) into the feedstock can cause a toxic overload, or excessive organic or solids content in the feedstock or insufficient retention time can cause an organic overload. These can lead to the process instability and potential digester failure, which can result in substantial downtimes and economic losses (Chen et al., 2012). Therefore, in order to use microbial-based strategies to manage anaerobic digesters, it is essential to distinguish between community shifts as part of a dynamic but stable system vs. a shift that can signal impending failure (Carballa et al., 2015).

Some studies have reported very distinctive microbial shifts in mesophilic anaerobic digestion during distress caused by different compounds. For example, the presence of copper sulfate (a potent anaerobic digestion inhibitor) caused a significant reduction of methanogens like *Methanosarcina* and several groups of microorganisms belonging to the order Clostridiales, including *Syntrophomonas*, *Butyrivibrio*, and *Caldicoprobacter*, combined with an enrichment of Methanobacteriales (e.g., *Methanobrevibacter* and *Methanosphaera*) and other bacterial groups (e.g., *Synergistetes*, Desulfobacterales, and *Mollicutes*) (Jordaan et al., 2019). Moreover, this disturbance also caused a reduction in microbial diversity and richness. Similarly, anaerobic digesters disturbed by the presence of phenolic compounds also showed the emergence of Methanobacteriales (i.e., *Methanoculleus*) at the expense of Methanosarcinales (Poirier et al., 2016) and the shift from the dominance of Clostridiales toward Bacteroidetes. Therefore, potential early indicators of digester failure could be a subtle decrease in the abundance of Methanosarcinales and the appearance of other methanogens belonging to the order Methanobacteriales and a surge of Bacteriodales combined with a drop in Clostridiales.

One of the common indicators of an early stage of digester failure is an increase in levels of intermediates products, especially propionate (Ahring et al., 1995). Propionate is generally metabolized into acetate by syntrophic bacteria in the presence of methanogens. Since propionate-degrading bacteria have different growth rates, different species are expected to dominate at higher propionate concentrations. Moreover, propionate can also be converted into biogas by hydrogenotrophic methanogens in syntropy with other organic acids-degrading bacteria (Han et al., 2020). Therefore, the emergence of these microbial species could serve as an early indicator of digesters disturbance. However, more studies are required to corroborate this and identify these potential microbial indicators. Overall, observing a transition in the microbial community is just a starting point for interpreting the alterations in digester behavior. This need to be correlated with reactor performance to better manage operational parameters and improve biogas production and stability.

The goals of this study were to evaluate the significance of changes in the microbial community of an anaerobic digester during failure caused by organic acid overloading, and during subsequent recovery. In order to increase the organic loading rate (OLR), and ultimately provide a shock-load leading to bioreactor failure, propionic acid was chosen to narrow the focus, and place targeted stress on the syntrophic relationships between propionate degraders and methane producers. The overall aim of this research was to identify a specific group, or groups, within the microbial community of an anaerobic digester that can be serve as early warning indicators of impending failure. Ultimately, these groups could then be specifically targeted during routine analysis of a digester, and a management strategy developed to circumvent failure and maintain steady operations.

## Materials and Methods

### Digester Operation and Sampling

A respirometer system was used to constantly monitor the biogas production from eight continuously stirred 0.5 L bioreactors set up as semi-continuously fed mesophilic (35°C) digesters. Sampling ports were used to feed fresh manure feedstock to semi-continuous bioreactors. Initially, the seed, collected from a bench-scale dairy manure digester, was combined in equal volatile solid (VS) ratio with the dairy manure feedstock, while a regular tri-weekly feeding with dairy manure maintained a 30-day hydraulic retention time (HRT). After 120 days of operation with an average organic loading rate (OLR) of 1.54 ± 0.07 kg VS/m^3^/d, all measured parameters indicated steady state operation, and the induced failure through the propionic acid overload was initiated. Two digesters were kept as controls being fed only manure at an OLR of 1.66 ± 0.12 kg VS/m^3^/d over the next 315 days. In the treatment (overloaded) digesters, propionic acid was added along with manure (in triplicate) to increase the starting OLR step-wise by 0.5 and 0.25 times, respectively, each week. This was continued for each digester until the biogas production decreased significantly, between 4 and 7 weeks, resulting in an OLR increase from 1.95 to 4.29 VS/m^3^/d (0.9–6.4 g/L reactor). After the addition of propionic acid was stopped, the digesters continued to be fed dairy manure at the same OLR as the control reactors until operational parameters recovered to similar levels as the controls. At the end, digestate was removed from the semi-continuous bioreactors through sampling ports, which were used to feed fresh manure feedstock.

The biogas production was monitored volumetrically with an automated flow-cell system (AER-200 Respirometer, Challenge Technology), while the manure and digestate pH was measured during feeding (Acorn pH 5, Oakton). Biogas composition (CH_4_, CO_2_, N_2_, and H_2_) was measured once a week using an Agilent 7890A GC. The total solids (TS) and VS were determined weekly for both manure and digestate using standard methods (APHA, 1995). The chemical oxygen demand (COD) was determined using a closed reflux colorimetric method (HACH Method 8000) and the alkalinity using standard methods (APHA, 1995). The volatile fatty acids (VFA) concentrations were determined after filtration (0.2 μm nylon, Whatman) based on a VFA standard 10 mM mixture (Supelco) using a Waters Breeze 2 HPLC system. Digestate samples were taken once a month for microbial analysis in sterile tubes (50 ml) and stored at −80°C until DNA extraction during the initial start-up and stabilization period (day -120 to 0), which was considered as the pre-shock stage. Digestate samples were taken once a week during the disturbance and recovery periods (day 0 to 315). All samples were sequenced for both bacterial and archaeal communities.

**FIGURE 1:**
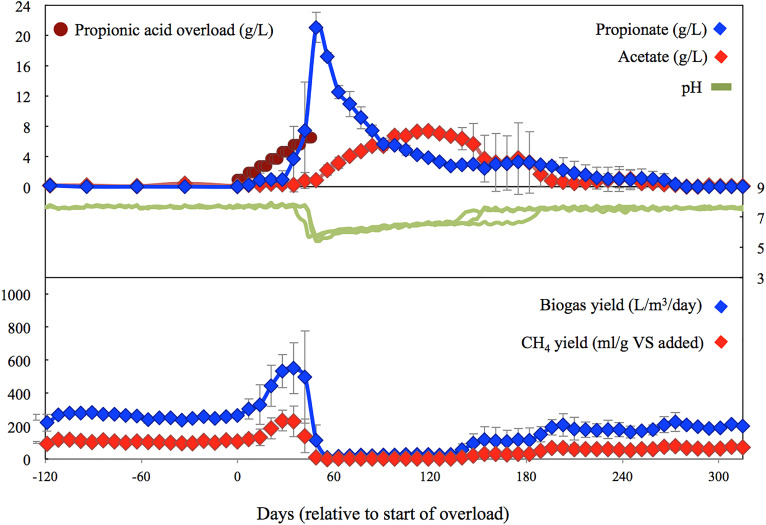
Average performance and biochemical characteristics for digesters spiked with propionic acid. Values during the last HRT are comparable with the control digesters (not shown), and similar to the initial performance.

In order to determine the early warning indicators using microbiome analysis, samples were taken through stable, failure, and recovery periods. Based on Figure 1, the microbial diversity was classified into six stages based on methane (CH_4_) production including: (1) Pre-shock stage, the initial steady period prior to propionic acid addition; (2) Pre-peak stage, the period between initial addition of propionic acid and peak in methane production; (3) Post-peak stage, the period between peak in methane production and no methane production; (4) Inhibition stage, the period with no evidence of methane production; (5) Recovery stage, the period when methane production was recovered till reached to a steady state; and (6) Steady state stage, the period when methane production resumed to levels similar to the Pre-shock stage.

### DNA Extraction and Quality Control

Approximately 200 mg of each sample was used for DNA extraction using the ZR DNA extraction Kit (Zymo Research Corp., Orange, CA, United States), which included a bead-beating step for the mechanical lysis of the microbial cells. DNA concentration was determined spectrophotometrically (NanoDrop 2000, Thermo Fisher Scientific). DNA purity was assessed by measuring A_260/280_ while DNA quality was evaluated by agarose gel electrophoresis following PCR amplification of the 16S rRNA gene using universal primers as previously described (Pullammanappallil et al., 2001).

### Library Construction and Illumina Sequencing

Library construction and Illumina sequencing were performed as described (Ventorino et al., 2018). Briefly, the V4 region of 16S rRNA gene was targeted for PCR amplification using modified F515/R806 primers (Caporaso et al., 2012), as previously described (Khafipour et al., 2009; Derakhshani et al., 2016a). Briefly, the reverse PCR primer was indexed with 12-base Golay barcodes allowing for multiplexing of samples. The PCR reaction for each sample was performed in duplicate and contained 1 μL of prenormalized DNA (20 ng/μL), 1 μL of each forward and reverse primers (10 μM), 12 μL high-quality reagents and chemicals (HPLC) grade water (Thermo Fisher Scientific), and 10 μL 5-Prime Hot MasterMix (5-Prime Inc., Gaithersburg, MD, United States). Reactions consisted of an initial denaturing step at 94 °C for 3 min followed by 35 amplification cycles at 94 °C for 45 sec, 50 °C for 60 sec, and 72 °C for 90 sec, and an extension step at 72 °C for 10 min in an Eppendorf Mastercycler pro (Eppendorf, Hamburg, Germany). PCR products were then purified using ZR-96 DNA Clean-up Kit (ZYMO Research) to remove primers, deoxyribosenucleotides (dNTPs), and reaction components. The V4 library was then generated by pooling 200 ng of each sample and quantified using Picogreen (Invitrogen, Burlington, NY, United States). This was followed by multiple dilution steps using pre-chilled hybridization buffer (HT1; Illumina, San Diego, CA, United States) to bring the pooled amplicons to a final concentration of 5 pM, measured by Qubit2.0 Fluorometer (Life technologies). Finally, 15% of PhiX control library was spiked into the amplicon pool to improve the unbalanced and biased base composition, a known characteristic of low diversity 16S rRNA libraries. Customized sequencing primers for read1 (5′-TATGGTAATTGTGTG CCAGCM GCCGCGGTAA-3′), read2 (5′-AGTCAGTCAGCCGGACTACH VGGGTWTCTAAT-3′), and index read (5′-ATTAGAWACC CBDGTAGTCCGGCTGACTGACT-3′; Integrated DNA Technologies, Coralville, IA, United States) were added to the MiSeq Reagent V2 Kit (300-cycle; Illumina). The 150 paired-end sequencing reaction was performed on a MiSeq platform (Illumina) at the Gut Microbiome and Large Animal Biosecurity Laboratories (Department of Animal Science, University of Manitoba, Winnipeg, MB, Canada). The sequencing data are uploaded into the Sequence Read Archive (SRA) of NCBI^1^ and can be accessed through accession numbers SRR12094968.

### Bioinformatics Analyses

Bioinformatics analyses were performed as described previously (Kosaka et al., 2008). Briefly, the PANDAseq assembler (Masella et al., 2012) was used to merge overlapping paired-end Illumina fastq files. The output fastq file was then analyzed using downstream computational pipelines in the open-source software package QIIME 1 v.9 (Caporaso et al., 2010). Chimeric reads were filtered using UCHIME (Edgar et al., 2011), and sequences were assigned to Operational Taxonomic Units (OTU) using the QIIME 1 implementation of UCLUST (Edgar, 2010) at 97% pairwise identity threshold using an open reference OTU picking process (Rideout et al., 2014). Taxonomies were assigned to the representative sequence of each OTU using an RDP classifier (Wang et al., 2007) and aligned with the Greengenes (v. 13.5) core reference database (DeSantis et al., 2006) using PyNAST algorithms (Caporaso et al., 2010).

### Alpha and Beta Diversity

Within-community diversity (α-diversity) was calculated by different indices of species richness and evenness including Chao1 and Shannon, using the open-source bioinformatics package QIIME 1 (Caporaso et al., 2010) and Phyloseq R package (3.1.0) (McMurdie and Holmes, 2013). The *p*-values were calculated, using the MIXED procedure of SAS (SAS 9.3) using a randomized factorial design where the effects of overload (control vs. propionic-spiked), methane-yield production stages (pre-shock, pre-peak, post-peak, inhibition, recovery, steady state), and their interaction were considered as fixed factors and the effect of manure as a random factor. Even depths of 10,000 and 500 sequences per sample were used to calculate the richness and diversity indices for bacterial and archaeal communities, respectively. The SAS MIXED procedure was used to calculate *p*-values and R- software for plotting the α-diversity graphs.

To assess the beta-diversity (β-diversity) differences among bacterial communities from different methane-yield stages within each induction model, non-metric multidimensional scaling (nMDS) ordination plots were generated using R software (3.1.1) by employing Bray-Curtis similarity matrices with a conventional cut-off of < 0.2 for the stress value (Munyaka et al., 2016). The resulting minimum stress solution was used to produce the nMDS plots, in which each data point represents one sample. The spatial distance between points in the plot was interpreted as the relative difference in the bacterial community composition; thus, points that were closer were more similar than points that were more distant. To assess the statistical differences in β-diversity of bacterial communities among control vs. propionic-spiked groups, permutation multivariate analysis of variance (PERMANOVA) (Anderson, 2005) was performed using the above-mentioned statistical model, and *p*-values were calculated.

### Clustering Analysis

To illustrate the distinct clustering pattern within each methane production stage in the propionic-spiked reactors, the relative abundance of the OTUs were binned into genus-level taxonomic groups and filtered to keep the most abundant genera found across all samples (cutoff value of greater than 0.1% of the community) (Derakhshani et al., 2016b). The resulting relative abundance table was normalized to correct for compositionality and also assist heat map-visualization of differentially abundant genera. The dissimilarity of samples were calculated based on Bray–Curtis measures using R “vegan” package (Oksanen et al., 2007) and the resulting matrix was subjected to unsupervised hierarchical clustering using R “dendextend” package (Galili, 2015) and visualized over the heat map of abundance matrix using R “complexheatmap” package (Gu et al., 2016). Genera were also clustered based on their Spearman’s correlation coefficient using R “complexheatmap” package.

### Correlation Coefficients

Associations between bacterial taxa with an abundance ≥0.1% of the community in the propionic-group, and archaeal taxa with an abundance ≥0.01% of the community were explored using non-parametric Spearman’s rank correlation implemented in PAST software (Hammer et al., 2001). For each correlation, correlation coefficient (Spearman’s Rho) and *p*-value were obtained (Wei and Simko, 2016) and the resulting correlation matrix was visualized in a heatmap format generated by the corrplot package of R (v. 02-0.2010)^2^. The correlation coefficient values ranged from -1 to +1 with larger absolute values indicating stronger relationship while positive and negative values indicating the direction of association. Alpha value for the correlation confidence intervals was set up as 0.05.

### Other Statistical Analysis

Normality of residuals for α-diversity was tested in the SAS UNIVARIATE procedure (SAS 9.3, 2012). Non-normally distributed data were log transformed. Original or transformed data were further analyzed using SAS MIXED procedure with the effects of overload (control vs. propionic-spiked), methane-yield stages (pre-shock, pre-peak, post-peak, inhibition, recovery, steady state) and their interaction as fixed factors and manure as a random factor. Tukey studentized range adjustment was used for all pairwise comparisons among the groups. GraphPad Prism (v. 6, GraphPad Software Inc. La Jolla, CA, United States) was used to plot the methane-yield production graphs using the multiple comparisons of two-way analysis of variance (ANOVA). Differences were reported as significant when *p* < 0.05 while trends were discussed at 0.05 ≤ *p* < 0.1.

## Results

### Effect of Propionic Acid Spike on the Methane Production

**FIGURE 2:**
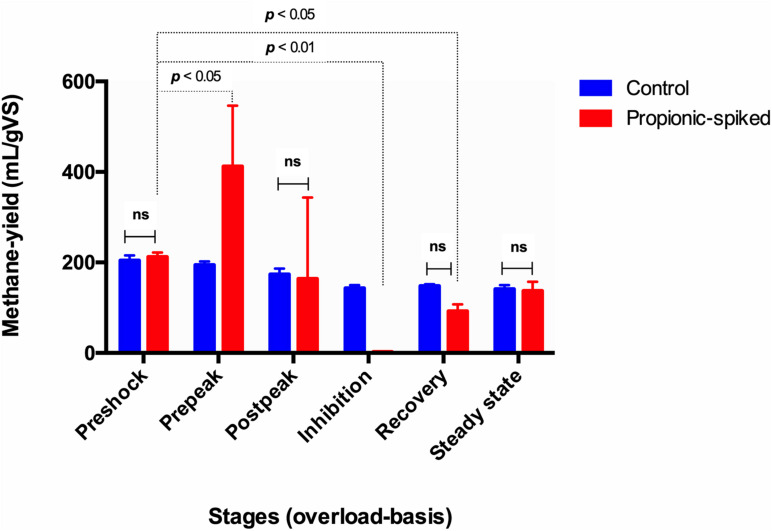
The comparison of different stages based on methane-yield production (ml/g) in both control and propionic-spiked reactors. Dotted lines indicate significant differences between the stages with the *p*-value shown in each case. There are no significant differences within different stages in control digesters (*P* < 0.05).

During the initial 120 days pre-shock stage, all five digesters performed similarly (Figures 1, 2), with the two controls averaging 500 ± 14 L biogas/m^3^ reactor/day, 204 ± 7 ml CH_4_/g VS added, a pH of 7.68 ± 0.05, and a total VFA concentration of 132 ± 66 mg/L. Over the following 315 days the performance of the controls remained fairly steady, with slight variations mainly induced by use of new batches of manure feedstock delivered from the dairy farm (every 2–3 months). The performance of the controls over the last 60 days averaged 409 ± 15 L biogas/m^3^ reactor/day, 138 ± 13 mg CH_4_/g VS added, a pH of 7.59 ± 0.04, and a total VFA concentration of 76 ± 36 mg/L. These values are comparable to the performance of the recovered digesters during the last 30 to 60 days.

As error bars in Figure 1 illustrate, the three digesters spiked with propionic acid reacted somewhat differently, failing after 4, 6, and 7 weeks, respectively, with the reactor that failed first accumulating the most VFAs and taking the longest to recover. The initial spikes of propionate were partially converted to acetate, with the remainder utilized after the acetate levels dropped due to the production of biogas. Up until the 6th spike, all of the initial propionate additions to the treatment digesters were consumed as observed by the increase in biogas/methane production. Overloading only occurred after this point, when the digester pH started to decrease to below 6, inhibiting methanogenesis. Digester recovery also coincided with the pH increasing back into the range of tolerance of the methanogens (i.e., above pH 6).

In addition to acetate and propionate, iso-butyrate, butyrate, iso-valerate, and valerate were also detected. The levels of these VFAs increased after failure from below detection to values between 163–265 mg/L, 929–1277 mg/L, 426–514 mg/L, and 607–666 mg/L, respectively. They remained at these levels until just before propionate levels started to decline. All of these changes in VFA (continual addition of propionic acid and accumulation of longer chain fatty acids) were echoed in the pH, which ultimately dropped to an average low of 5.87 ± 0.41, corresponding to the period of methanogenic inactivity.

As seen in Figure 2, the control digesters exhibited similar methane yields throughout the entire study. In the propionic-spiked digesters, there was a significant increase during the pre-peak stage (*p* = 0.01) corresponding to the addition of readily usable substrate and indicating the presence of a robust population of syntrophic bacteria. As the shock load of propionate increased, there was an eventual collapse in methanogenesis, which we refer to as the inhibition stage (*p* = 0.001). There is no significant difference between pre-shock stages in the control vs. the propionic-spiked group (*p* = 0.81), as well as no significant difference between all stages in the control group. Hence, both the pre-shock stage as well as the control digesters will be used to establish a baseline microbial community for steady state operation, which can be compared to the communities of the propionic-spiked digesters.

### Effect of Propionic Acid Spike on Alpha-Diversity of the Microbial Population

**FIGURE 3:**
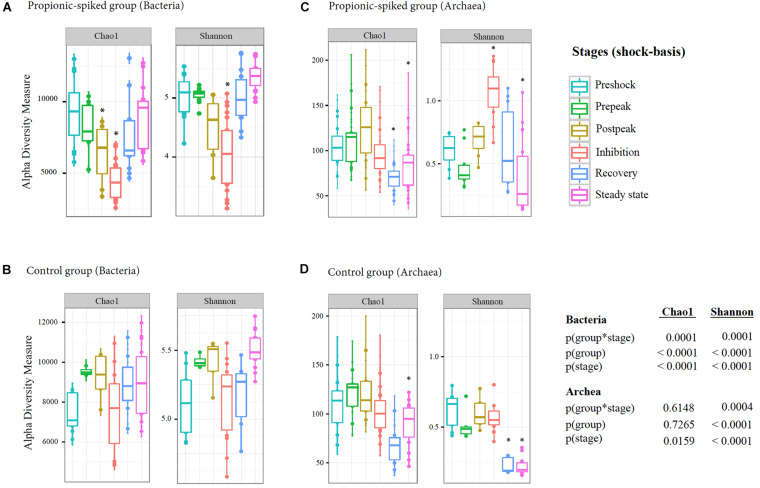
Summary of α-diversity indices within different stages of methane-yield production in propionic-spiked **(A,C)** and control **(B,D)** sample reactors. The graphs indicate the comparison of Chao1 and Shannon indices of α-diversity in both bacterial **(A,B)** and archaeal **(C,D)** communities. Y-axis shows α-diversity measure and X-axis of each graph shows different stages (shock-basis), each colorful dot represents sample taken during each stage. *: Significant difference between each marked stage and pre-shock stage. Statistical analyses were conducted using MIXED procedure of SAS (*P* < 0.05).

Comparing the microbial community in the control vs. propionic-spiked group, there was no significant difference (*p* = 0.80) during the pre-shock stage, while there was a decreasing trend in Chao1-index of α-diversity (*p* = 0.09). This was followed by a significant drop during the post-peak stage (*p* = 0.03) and continued to decrease during the inhibition stage (*p* = 0.01) (Figure 3A). Subsequently, after the inhibition stage, the average Chao1-index significantly increased from 3534.6 to 5671.3 and Shannon-index from 5.81 to 7.25 during the recovery stage (*p* = 0.02). As the Chao1 and Shannon graphs of bacterial community indicate, within the control group of bacterial community (Figure 3B), there was no significant difference in all stages. Both Chao1 and Shannon indices at the recovery and steady state stages stayed at similar levels with the pre-shock stage (*p* = 0.6), which indicates the eventual alleviation of the bacterial dysbiosis caused by the ever-increasing addition of propionic acid to the anaerobic digesters.

With respect to the archaeal community, there was an increasing trend in Chao1 and Shannon index until the inhibition stage in the propionic-spiked reactors (Figure 3C). There were significant decreases in species richness during the recovery (*p* = 0.02) and steady state stages (*p* = 0.04) showing by Chao1-index compared to the pre-shock stage (Figure 3C). In contrast, propionic-overload increased the diversity of archaea during inhibition stage as indicated by Shannon-index. There was also a significant decrease in species richness and diversity in the control group of the archaeal community at steady state compared to the pre-shock stage (*p* = 0.03) (Figure 3D), while no difference was observed between the control and propionic-spiked groups during steady states. Therefore, the α-diversity indicates the existence of microbiome dysbiosis during the post-peak period of propionic acid overload. The ultimate recovery of methane production during the recovery stage indicates the reformation of key members of both bacterial and archaeal communities.

### Effect of Propionic Acid Spike on Beta-Diversity

**FIGURE 4:**
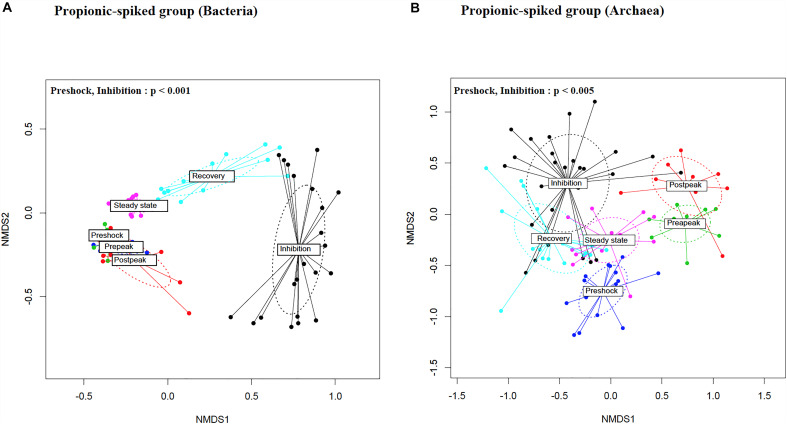
Non-metric multidimensional scaling (nMDS) ordination plot, a measure of relative dissimilarities in the **(A)** bacterial and **(B)** archaeal communities in the propionic-spiked sample reactors. The colored points are shaded according to different stages. The *p*-values were calculated using PERMANOVA. For the multiple-comparison tests, only the significant *p*-values were included. Trends were discussed at *p* < 0.1.

To compare bacterial and archaeal diversity between different stages within the propionic-spiked group and the control group, nMDS plots based on Bray-Curtis dissimilarity matrices were generated (Figures 4A,B, respectively). The results indicate an overlap between all stages in the control group. Likewise, the pre-peak and post-peak overlapped with the pre-shock community, confirming the resilience of the bacterial community to the initial addition of propionate. A distinct clustering, however, was observed between the inhibition and pre-shock stages within the propionic-spiked group showing the highest diversion for both bacterial (*p* = 0.002) and archaeal (*p* = 0.01) communities. With respect to bacterial community, samples from the recovery and steady state stages were clustered closely toward the pre-shock stage. A distinct pattern was observed in the archaeal samples of the propionic-spiked group. These shifts in clustering patterns of microbial community composition corresponded well to the changes of methane-yield (Figure 2), indicating an adaptation of a more favorable methanogenic community in response to the addition of propionate, and higher rate of methanogenesis. Once methanogenesis was completely inhibited, distinctive 16S rRNA gene of archaea remained. During recovery, the archaeal community composition shifted back toward the original composition. Overall, the propionic acid spike altered β-diversity in bacterial and archaeal communities as suggested by 16S rRNA gene, compared to the pre-shock stage, whereas the inhibitory effect was slowly overcome during the recovery stage.

### Clustering of Microbial Communities in Propionic-Spiked Reactors

**FIGURE 5:**
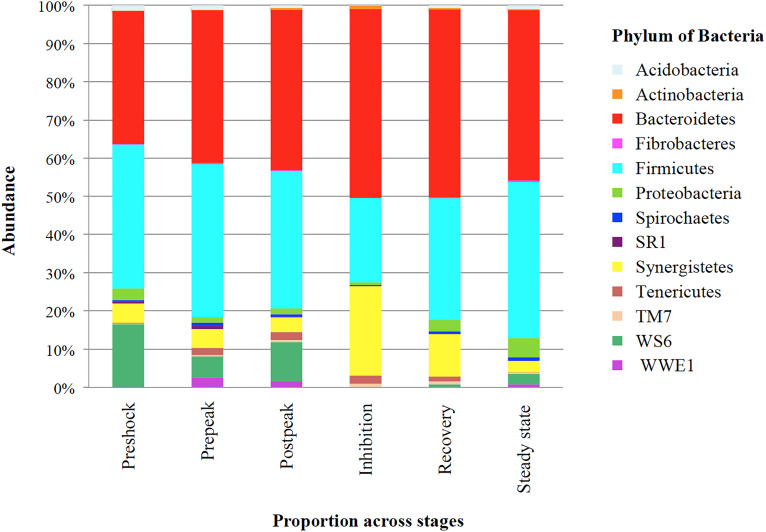
The proportion of bacteria phylum in different stages based on propionic overload. Each bar represents the phylum of abundant phylum (>1% of community) (*Y*-axis) in correspond to each stage (*X*-axis) of propionic-spiked reactors. Pre-shock samples are considered as control, due to zero bacterial and archaeal community differences with each other.

Initially, as Figure 5 illustrates, the most relative abundant phylum (>1% of community) in propionic-spiked sample reactors, were Firmicutes and Bacteroidetes. After propionic overload during the inhibition stage, the proportion of Firmicutes decreased from 36.02 to 21.26% compared to the pre-shock stage, whereas the proportion of Bacteroidetes increased from 33.81 to 47.89% compared to pre-shock stage. Most striking is the decrease of the members of the 3rd most dominant candidate phylum, WS6, which went from >10% in the stages prior to inhibition to almost undetected during the inhibition phase, only to recover somewhat in steady state. Conversely, the phylum, Synergistetes, reached >20% of the community during the inhibition only to return pre-shock values of <5% in steady state.

**FIGURE 6:**
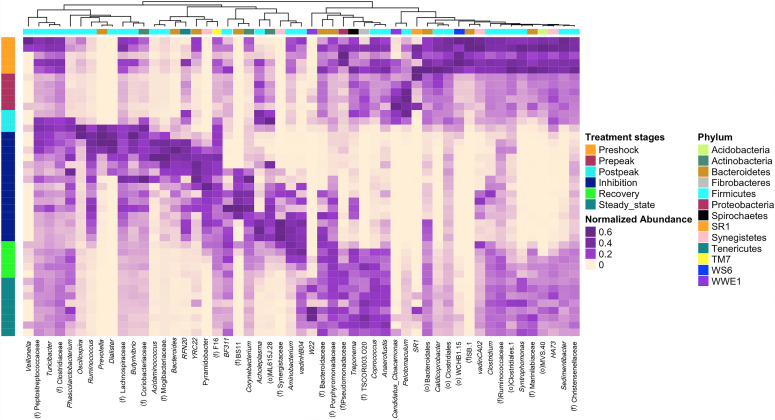
The clustering pattern of bacterial community of control and propionic-spiked sample reactors. Rows correspond to samples and columns correspond to abundant genera (>0.1% of community). The “normalized abundance” key relates colors to the normalized proportions of genera (relative abundance of each genus divided by the Euclidean length of the column vector). The left dendogram shows how samples are clustered based on their Bray–Curtis dissimilarities (using unweighted pair group method with arithmetic averaging UPGMA). The significance of clustering patterns has been calculated based on 9,999 permutations and *p*-values calculated based on PERMANOVA. The top dendogram shows how genera correlate (co-occur) with each other based on their Spearman’s correlation coefficient. The “Phylum” key relates the top annotations to the corresponding phylum of each genus. The “Stages,” key relates samples to the propionic-spiked group different stages based on methane-yield production (Pre-shock, Pre-peak, Post-peak, Inhibition, Recovery, Steady state). Color codes have been also used to highlight bacterial genera that were significantly associated with each stage of propionic-spiked group (*P* < 0.05).

**FIGURE 7:**
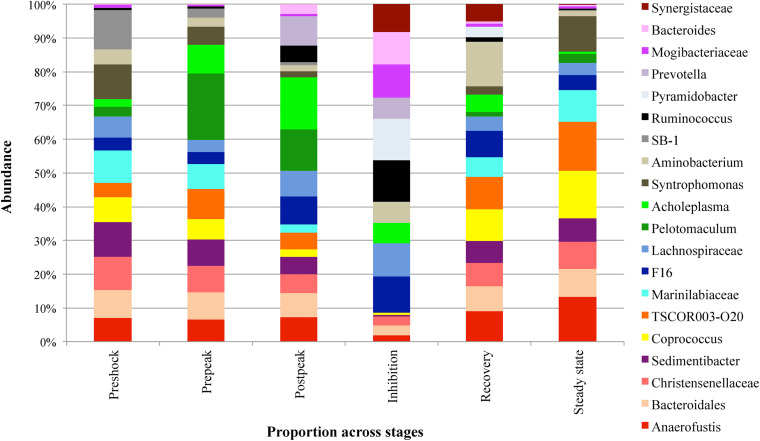
The comparison of different abundant genera (>0.1% of community) with significant abundance difference in different stages of propionic-spiked reactors. A statistical difference were evaluated by LEfSe, a metagenome analysis approach which performed the linear discriminant analysis following the Wilcoxon Mann–Whitney test to assess effect size of each differentially abundant variable.

Next, a clustering analysis based on Bray-Curtis dissimilarity was employed in R (Gu et al., 2016) to investigate which phyla and genera were correlated to biodigester failure and whether the clustering pattern of microbiota at the genus level changed following the propionic acid overload. As Figure 6 illustrates, the post-peak and inhibition stages exhibited a distinctly different microbial compositions compared to the other stages. Following up on the previous analyses, using LEfSe and MIXED procedure of SAS, the most significant changes in the relative abundance of bacterial taxa were identified (Figure 7). At the inhibition stage, a significant increase in relative abundance of Lachnospiraceae, Mogibacteriaceae, and *Ruminococcus* from Firmicutes phylum, *Bacteroides* and *Prevotella* from Bacteroidetes phylum, and *Pyramidobacter* from Synegistetes phylum. In contrast, at the inhibition stage, there was a significant absence of *Coprococcus, Syntrophomonas, Anaerofustis*, Christensenellaceae, and *Sedimentibacter* from Firmicutes, *TSCOR003.O20* from Fibrobacteres, and Marinilabiaceae, and *Bacteroidetes* from Bacteroidetes. At the recovery and steady state stages, there were clear alterations in the clustering pattern of aforesaid taxa, indicating the vitality of their presence for methane production process.

**FIGURE 8:**
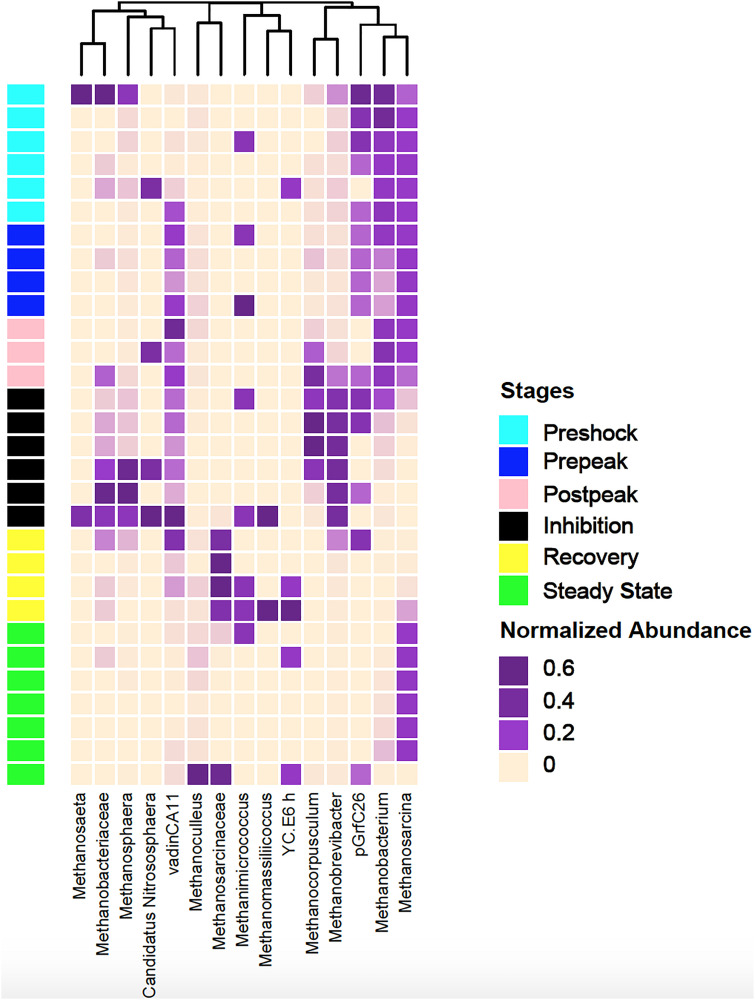
The clustering pattern of archaeal community of control and propionic-spiked sample reactors. Rows correspond to samples and columns correspond to abundant genera (>0.1% of community). The “normalized abundance” key relates colors to the normalized proportions of genera (relative abundance of each genus divided by the Euclidean length of the column vector). The top dendogram shows how genera correlate (co-occur) with each other based on their Spearman’s correlation coefficient. The “Stages” key relates samples to the propionic-spiked group different stages based on methane-yield production (Pre-shock, Pre-peak, Post-peak, Inhibition, Recovery, Steady-state).

The most significant changes in the relative abundance of archaeal taxa were identified and shown in Figure 8. The two archaeal taxa that exhibited a significant reduction in their relative abundance during the inhibition stage were *Methanosarcina* and *Methanobacterium.* They regain their abundances following the recovery stage and into the final steady state stage. The relative abundances of 16S rRNA gene from *Methanobrevibacte*r, *Methanosphaera, and Methanocorpusculum* increased (from 17.36 to 79.45%, from 0.14 to 1.12%, and from 0.09 to 0.6%, respectively) during the inhibition stage, indicating that these organisms were introduced through the continual addition of manure, but remained inactive as no methane production was observed. Their relative abundances decreased significantly (from 91.71 to 12.14% and from 2.98 to 0.73%, respectively) during the recovery and state-state stages (*p* = 0.01) and as replaced by *Methanosarcina* and *Methanobacterium* as conditions for methanogenesis improved.

### Correlation Analysis

The non-parametric Spearman’s rank correlation analysis showed significant associations between the several bacterial taxa ≥0.1% and archaeal taxa ≥0.01% of the communities in the propionic acid-spiked reactors. During the post-peak stage, a significant negative correlation was observed between reduction in *Syntrophomonas* and increase in *Methanocorpusculum* in all propionic-acid spiked reactors. As the propionic-acid spiked digesters recovered, *Syntrophomonas* increased, which was positively correlated with *Methanosarcina* and negatively correlated with *Methanobrevibacter*. Other bacterial taxa that were positively correlated to *Methanosarcina* but negatively correlated to *Methanobrevibacter* during the recovery stage included Marinilabiaceae, *Sedimentibacte*r, Christensenellaceae, and Bacteriodales. During recovery, the reduction in the abundance of Lachnospiraceae was positively correlated to *Methanobrevibacter* but negatively correlated with a *Methanosarcina, Methanobacterium*, and *Methanoculleus*.

## Discussion

The anaerobic digestion process involves four categories of microogranisms, hydrolyzers, acidogens, acetogens, and methanogens (Franke-Whittle et al., 2014). Due to syntrophic relationships among these groups, a dramatic shift in one group (e.g., over-/under-expressed populations and functions) can lead to a dysbiosis-related anaerobic biodigester failure. Any changes in α- and β-diversity can be considered as a characteristic of dysbiosis during biodigester failure. In order to study the mechanisms underlying anaerobic digestion failure, we used propionic acid to overload the experimental biodigesters over time leading to the digester failure. Therefore, our aim was to provide insights into potential microbiota-based biomarkers as early warning indicators of impending bioreactor failure due to VFA accumulation and subsequent pH decrease.

As illustrated in Figure 3, using the within-communities Chao1 and Shannon-index of α-diversity, there was a significant decrease in species richness during the post-peak and inhibition stages, compared to the pre-shock stage in both bacterial and archaeal communities. This can be considered as the first dysbiotic signature that leads to methane production failure. Jordaan et al. (2019) studied the addition of copper to the dairy manure digesters as another cause of disturbance and reported similar reduction patterns in diversity and richness indices. This was followed by a recovery of the community complexity after the inhibition period ended.

In addition to α-diversity, comparing diversity between different stages within propionic-spiked and control groups, using nMDS plots of β-diversity, there was a distinct clustering pattern during overload followed (by its highest diversion at the inhibition stage) in both archaeal and bacterial communities. Furthermore, as shown in Figures 4A,B, there is a shift back at the recovery and steady state stages toward pre-shock conditions. Similarly, both bacterial and archaeal communities experienced a distinct shift during copper addition (a methanogenic community inhibitor), with a shift back during recovery stage toward original community composition (Jordaan et al., 2019). In our experiment, similar to the results of several other studies with anaerobic biodigesters, Firmicutes and Bacteroidetes were the most dominant phyla (Figure 5; Sundberg et al., 2013; De Francisci et al., 2015; Jordaan et al., 2019). As Hanreich et al. (2013) described, some members of Firmicutes such Ruminococcus are among the main cellulose degraders. Several members of Bacteroidetes, e.g., *Prevotella* are also associated with the digestion of fiber (Hanreich et al., 2013). Bacteroidetes, as saccharolytic and sugar fermentative bacteria generating propionate and acetate in anaerobic bioreactors (Sundberg et al., 2013), were in high relative abundance during the start-up and overloading periods (Sundberg et al., 2013; Martin Vincent et al., 2018). Members of the Candidate phylum Dojkobacteria (WS6) were the third most abundant group prior to the addition of propionate. While their function in the digester is still unknown, bacteria from this phylum reported to be found in high numbers in several sludge digesters (Wrighton et al., 2016). On the basis of genome sequencing of representatives of this candidate phylum, these small genome organism (<1 Mb) lack many key pathways, and are suggested to be either syntrophic or parasitic to other bacteria anaerobic (Wrighton et al., 2016). In the present system, they were one of the last groups to recover after steady state was reached.

Among shifts in bacterial composition during propionic-acid overload were the increase of *Pelotomaculum* from the Firmicutes phylum. *Pelotomaculum*, a member of known propionate oxidizing bacteria (POB) like *Syntrophomonas* and *Smithella*, is able to convert propionate to acetate, H_2_, and CO_2_ (Kosaka et al., 2008; Liu and Lu, 2018). Many recent studies indicated *Pelotomaculum* as the dominant POB group in anaerobic digestion systems (Shigematsu et al., 2006; Ban et al., 2013). In a recent study by Li et al. (2014), *Pelotomaculum* sp. comprised 1.2% of the total prokaryotes in manure-straw co-digestion experiments and was considered the dominant POB. A positive correlation between the increased proportion of *Pelotomaculum* sp. and *Methanoculleus* sp. was also observed (Shigematsu et al., 2006; Li et al., 2014; Li C. et al., 2015). As discussed by Hori et al. (2006) *Methanoculleus* sp. was characterized not only as the ideal methanogenic partners for syntrophic propionate oxidation but also for syntrophic acetate oxidation (Hori et al., 2006). The increased abundance of *Pelotomaculum* in the pre-peak stage (10.33–34.48) is followed by its depletion after the post-peak stage, during the inhibition stage (20.97–0.04) and its slow recovery during the recovery stage (0.04–0.2) (Figure 6). *Methanoculleus* showed a slight abundance increase during the pre-peak stage (0.13–0.54), and then dropped after the peak (0.54–0.01) during inhibition stage, which illustrates the role of a positive correlation between *Pelotomaculum* and *Methanoculleus* in propionic digestion.

A member of Fibrobacteres phylum, *f*.*TSCOR003.O20* increased significantly after the initial addition of propionate, however, it was depleted at the inhibition stage, and then re-appeared in the recovery stage. Based on Figure 7, a similar trend can be observed in *Candidatus cloacamonas* from WWE1, however, without any recovery during recovery or steady state stages. Also, an acid-producing bacterium, *Acholeplasma*, increased after the shock-load, decreased in abundance during first weeks of inhibition, re-appeared sooner than other bacteria before the recovery stage, and again decreased during the final steady state period. Similar to reports by Li L. et al. (2015), the significant growth of acid-producing bacteria results in the accumulation of VFA, which is known as a crucial factor in anaerobic digester failure (Li L. et al., 2015). Martin Vincent et al. (2018) and others reported VFAs as indicator for monitoring the anaerobic digestion process (Franke-Whittle et al., 2014; Martin Vincent et al., 2018). As observed in this study, between the three propionic-spiked digesters, the reactor that failed first was accumulating the most VFAs and taking the longest to recover. Also, the initial spike of propionate increased methane. Upon further spikes, propionate decrease was associated with an increase of acetate levels, but without further biogas production, with the remainder utilized only after the acetate levels had dropped in association with recovery and renewed methanogenesis.

A significant negative correlation (*p* < 0.03) between decreased abundances of *Syntrophomonas* and *Methanocorpusculum* during the post-peak stage was observed. *Syntrophomonas*, which is a recognized POB, significantly decreased during the post-peak stage and then recovered during the recovery and final steady state stages. As Ventorino et al. (2018) indicated, *Methanocorpusculum* has a negative correlation with pH, leading to the presence of this archaea at the low pH (such as during the of post-peak stage) in anaerobic digestion processes (5 < pH < 6). Additionally, Savant et al. (2002) reported higher abundance of *Methanobrevibacter* at pH <6, which also corresponds to our observation after the propionic acid shock. Goux et al. (2015) reported that at low pH the archaeal populations shifted from acetotrophic to hydrogenotrophic methanogenesis. Jordaan et al. (2019) also reported that *Methanobrevibacter* and *Methanosphaera*, the two prominent archaea associated with the rumen, and *Methanocorpusculum* a dominant archaea in manure, increased in relative abundance during the inhibition stage after copper sulfate addition (Jordaan et al., 2019). On the other hand, *Methanosarcina* and *Methanobacterium* (dominant digester archaeas) were reduced in abundance during inhibition. De Vrieze et al. (2012) stated that the decrease in abundance of *Methanosarcina* sp. associated with an increase in *Methanobrevibacter* and *Methanosphaera* could be a potential warning signal of future reactor failure. In a continues-feed system (such as the one used in this study), as manure rich in *Methanobrevibacter* and *Methanosphaera* is fed into an inhibited digester, the relative abundance of manure-based archaea to digester-based archaea (i.e., *Methanosarcina* sp.) will steadily increase.

Furthermore, during the inhibition stage, the shift in the archaeal community was associated with microbial community changes. For instance, *Prevotella* of Bacteroidetes, increased in relative abundance at the fastest rate of any bacteria, which indicates that it could be the most resilient species under high VFA and low pH conditions. However, with the continual addition fresh manure, an increase in relative abundance of other groups such as *Bacteroides*, Lachnospiraceae, Mogibacteriaceae, *Ruminococcus*, and *Pyramidobacter* expected to be coming from the manure (Ozbayram et al., 2018) was observed at later time points during inhibition, which could have led to competition for nutrients with *Prevotella*, resulting in depletion of *Prevotella*. At later time points during inhibition, there was a significant increase in other bacteria such as Synergistaceae, *Acholaplasma*, and *Aminobacterium*, which resulted in depletion of the above-mentioned five bacteria.

*Aminobacterium* as anaerobic amino-acid degrading bacteria are a member of the Synergistetes phylum, involved in acidogenesis (Diaz et al., 2006; Nelson et al., 2011). They are accompanied by *Acholaplasma*, which are also acid-producing bacteria, and therefore involved in the increased VFA production. However, as Figure 7 illustrates, their resilience appeared to be less than other organisms, as their dominance was short-lived during the recovery stage.

During the inhibition stage, some genera completely disappeared. While some recovered during the recovery stage, others remained at very low abundance. Amongst these bacteria, *Anaerofustis* from the phylum of Firmicutes appeared sooner than others, which could show their potential for surviving in a higher range of VFA. Although their presence does not show a healthy overall state, they could be indicators of acidogenesis and augmented methanogenesis.

Our data suggest a healthy state of an anaerobic biodigester can be linked to the presence of *TSCOR003.O20* from Fibrobacteres, Marinilabiaceae from Bacteroidetes, *Sedimentibacter* and *Syntrophomonas* from Firmicutes, and the absence of *Bacteroides* from Bacteroidetes, Mogibacteriaceae, Lachnospiraceae, and *Ruminococcus* from Firmicutes, and *Pyramidobacter* from Synegistetes, organisms which are more typically associated with manure (Ozbayram et al., 2018; Flores-Orozco et al., 2020). Marinilabiaceae from Bacteroidetes, as obligatory anaerobic and saccharolytic bacteria (Hanreich et al., 2013), were reported to be abundant in ammonia-rich chicken-waste anaerobic digesters (Martin Vincent et al., 2018). The significant drop in Marinilabiaceae during the post-shock stage could be used as an early indicator for reactor failure. An increase of *Bacteroides*, which secrete hydrolyzing enzymes such as cellulases, amylases, proteases and lipases, and play an important role in the acidogenesis phase, could serve as warning signal to impending reactor inhibition (Martin Vincent et al., 2018). *Sedimentibacter* species are able to ferment glycine to acetic acid without H_2_ gas production (Lin et al., 2018). Lachinospiraceae and *Ruminococcus* with the capability of degrading complex vegetable materials to short-chain fatty acids, specifically acetate, propionate, and butyrate (Cardinali-Rezende et al., 2016) were reported to appear simultaneously with Mogibacteriaceae in anaerobic digesters (da Silva Martins et al., 2017). Also, bacteria belonging to Ruminococcaceae utilize ammonia, produced by the biological degradation of nitrogenous compounds, primarily proteins, as a source for nitrogen (Cardinali-Rezende et al., 2016; da Silva Martins et al., 2017). Zhu et al. (2018) reported the ability of Lachnospiraceae in secreting endoglucanases and a glycosyl hydrolase enzyme in breaking down complex lignocellulosic biomass (Zhu et al., 2018).

Some key organisms to look for as the first warning signs of change would be expected to be those most sensitive to the addition of the VFA early in the pre-peak period despite the continued or even enhanced methanogenesis observed. Some of these same organisms reappear early in the recovery phase and form an early warning system that the recovery is underway (Figure 6). These bacteria appear to rebuild their populations in conjunction with the appearance of the first acetoclastic methanogens of the Methanosarcinaceae. It is unclear if the new steady state observed would eventually match the pre-shock state. Different microorganisms can lead to the same functional steady state (Figure 4). Indeed, dominant organisms pre-shock such as the representative of the order *WCHB1.15* from the Dojkabacterial phylum (WS6) (Figure 6), did not recover to their previous abundance at the end of the experimental monitoring, while the representative of family SB.1, a major organism pre-shock, was not observed at the final steady state.

Altogether, the occurrence of propionic acid overload in anaerobic biodigesters leads to substantial shifts in the microbial community composition, triggering changes to significant members of bacterial and archaeal communities, simultaneously. The shifts in both communities due to sensitivity to the overload give rise to VFA accumulation and corresponding pH drop, resulting in long-term methane cessation and biodigesters failure. Although, quite different in composition and diversity, the re-assemblance of archaeal and bacterial communities at the final steady state and corresponding resumption of biogas production, highlights the biodigester’s microbial resilience and recovery potential.

## Conclusion

This study demonstrated that the occurrence of a propionic acid overload in manure biodigesters led to a severe failure due to the accumulation of VFA, which caused a drop in pH and ultimately, the loss of biogas production. The bacterial and archaeal communities underwent some distinctive shifts after the propionic acid shock and during the inhibition stage. Nevertheless, after failure, both bacterial and archaeal compositions at steady state returned to similar levels of their pre-shock stage, highlighting the digester’s microbial resilience and recovery potential. The subtle changes in the microbial compositions during the organic shock could serve as a potential early indicator of the digester’s distress.

## Data Availability Statement

The raw data supporting the conclusions of this article will be made available by the authors, without undue reservation.

## Author Contributions

AK: writing-original draft preparation, investigation, formal analysis, and visualization. EJ: experimental design and data collection. DF-O: review and editing. EK, DL, and RS: review, editing, and conceptualization. NC: resources, writing—review and editing, supervision, funding acquisition, methodology, and conceptualization. All authors contributed to the article and approved the submitted version.

## Conflict of Interest

The authors declare that the research was conducted in the absence of any commercial or financial relationships that could be construed as a potential conflict of interest.

## Footnotes

1 http://www.ncbi.nlm.nih.gov/sra

2 https://cran.r-project.org/
